# Ultrastructural characterization of neuropeptide Y synapses in the central inferior colliculus of the Fischer Brown Norway rat

**DOI:** 10.1016/j.npep.2025.102566

**Published:** 2025-10-13

**Authors:** Laila S. Almassri, Joshua C. Harris, Kristen M. Crane, Andrew P. Ohl, Nick J. Tokar, Brett R. Schofield, Jesse W. Young, Jeffrey G. Mellott

**Affiliations:** a Department of Anatomy and Neurobiology, Northeast Ohio Medical University, Rootstown, OH, USA; b University Hospitals Hearing Research Center, Northeast Ohio Medical University, Rootstown, OH, USA

**Keywords:** Central inferior colliculus, NPY, Synapse, Ultrastructure

## Abstract

**Introduction:**

The central nucleus of the inferior colliculus (ICc) is the primary target for ascending auditory projections from the lower auditory brainstem nuclei and the main source of ascending projections to the auditory thalamus. The ICc has an extensive network of tonotopically organized neurons, whose classification is a subject of broad interest. In order to understand the extensive microcircuitry of the ICc, it is important to ascertain the synaptic arrangements among molecularly identified neuronal populations. The objective of this study was to describe inhibitory Neuropeptide Y (NPY) synapses in the central nucleus of the IC.

**Method:**

We used electron microscopy (EM) and post-embedding anti-NPY immunogold histochemistry on tissue from adult Fischer Brown Norway rats. Inhibitory NPY synapses were identified by pleomorphic vesicles, symmetric synaptic junctions, and NPY-immunopositive presynaptic boutons.

**Results:**

The data demonstrate that NPY terminals largely (~75 %) form symmetric synapses with pleomorphic vesicles, mostly target small and medium dendrites, and have larger average bouton areas, active zone lengths, and vesicle pools, as compared to the overall GABAergic population.

**Discussion:**

The subset of GABAergic neurons that co-express NPY feature synaptic characteristics suggesting that NPY neuromodulation has a widespread role in regulating excitation in the ICc.

## 1. Introduction

The auditory system is an essential sensory modality that enables hearing, communication, and processing of information. Auditory neurons routinely encode spectral, spatial, and temporal properties of sound and relay this information via highly divergent circuits (Winer and Schreiner, 2005). The inferior colliculus (IC) is the largest auditory nucleus that houses the most diverse collection of bidirectional pathways in the auditory system (Winer and Schreiner, 2005; Ito et al., 2016; Oliver et al., 1997; Cant, 2005; Oliver, 2005; Schofield, 2005; Ito and Malmierca, 2018). The IC is commonly demarcated into three anatomical subdivisions: the central nucleus (ICc), the dorsal cortex (ICd), and the lateral cortex (IClc) (see reviews: Oliver, 2005; Syka, 2020). Ascending auditory pathways from lower brainstem nuclei project to the ICc and converge onto ICc cells (Sivaramakrishnan and Oliver, 2001; Kelly and Caspary, 2005; Oliver, 2005; Saldaña and Merchán, 2005; Schofield, 2005; Wu, 2005; Cant and Benson, 2006). The convergence of inputs from different origins combined with the intrinsic physiological properties of the target cells determines their response properties and thus their contributions to sensory processing (Sivaramakrishnan and Oliver, 2001; Kelly and Caspary, 2005; Oliver, 2005; Saldaña and Merchán, 2005; Cant and Benson, 2006). However, the exact inputs onto molecularly distinct cell types remains unclear. Understanding the synaptic connections is an important step towards deciphering IC circuitry and categorizing IC neurons.

The inhibitory neuronal population in the IC expresses GABA and represents approximately 20–25 % of cells in each subdivision, while the excitatory population expresses glutamate and represents the remaining 75–80 % of cells (Oliver et al., 1994; Merchán et al., 2005; Ito et al., 2011; Ito, 2020; Mellott et al., 2014a, 2014b; Ouda et al., 2015; Beebe et al., 2016). Across many brain regions, GABAergic neurons are divided into distinct functional groups based on their neuropeptide co-transmission (Hof et al., 1999; Kubota and Kawaguchi, 1994). Recent reports have focused on characterizing a subset of GABAergic neurons in the IC that co-express Neuropeptide Y (NPY) (Silveira et al., 2020, 2023, 2024; Anair et al., 2022; Almassri et al., 2025). NPY is a 36-amino acid peptide that is abundant throughout the brain and whose functions include modulating neuronal excitability, neuroplasticity, and memory (Malva et al., 2012; Herzog, 2012; Gøtzsche and Woldbye, 2016; Suyama and Yada, 2018; Silveira et al., 2023). NPY neurons in the IC have a stellate morphology with dendritic arbors reaching across iso-frequency laminae (Silveira et al., 2020). Targets of their projections include the contralateral IC and the auditory thalamus (Nakagawa et al., 1995; Silveira et al., 2020). NPY is co-expressed by a subset of the GABAergic cells in the IC, and more specifically ~60 % in the ICd, ~50 % in the ICc, and ~ 35 % in the IClc (Silveira et al., 2020; Almassri et al., 2025). Our previous study demonstrated that NPY cells have average profile areas ranging from 218 to 301 μm^2^, which falls into the medium sized GABAergic cell category (Ono et al., 2005; Ito et al., 2009; Roberts and Ribak, 1987a, 1987b; Oliver, 2005; Beebe et al., 2016; Koehler et al., 2023; Almassri et al., 2025). Broadly, IC GABAergic soma sizes and neuropeptide co-expression are linked to their neurochemical profiles and circuitry (Ito et al., 2009; Beebe et al., 2016; Goyer et al., 2019; Ito, 2020; Silveira et al., 2020, 2023; Kreeger et al., 2021).

A pertinent step in understanding IC circuitry is identifying cell to cell synaptic connections. Electron microscopy (EM) provides the needed resolution to ascertain detailed synaptic connections at the ultrastructural level. Synaptic features enable us to differentiate between excitatory and inhibitory synapses, presynaptic bouton area can provide clues into the origins of the inputs, and the spatial arrangement of synapses on dendrites, spines, boutons, and somata is critical for synaptic integration. Characterizing these features is fundamental to our understanding of how IC neurons process and relay information.

This report builds on our previous descriptions of GABAergic synapses in the ICc (Wawrzyniak et al., 2025). In those studies, we examined the ultrastructure of GABAergic synapses across the tonotopic ICc axis and demonstrated that at young age (3–4 months) GABAergic synaptic density, terminal areas, active zone lengths, and vesicle pools were analogous across the tonotopic ICc axis (Wawrzyniak et al., 2025). Furthermore, the results agreed with previous reports that GABAergic terminals across the ICc form symmetric synapses, contain pleomorphic shaped vesicles, and largely (~85 %) target dendrites (Wawrzyniak et al., 2025; Rockel and Jones, 1973; Oliver and Morest, 1984; Ribak and Roberts, 1986; Roberts and Ribak, 1987a; Paloff and Usunoff, 1992; Helfert et al., 1999; Nakamoto et al., 2013, 2014; Mafi et al., 2022; Mellott et al., 2024).

To gain a deeper understanding of the subset of GABAergic neurons that co-express NPY in the auditory midbrain, the current study sought to examine the ultrastructure of NPY synapses in the ICc using EM and post-embedding anti-NPY immunogold histochemistry on tissue from adult Fischer Brown Norway rats (2–3 months old). We demonstrate that NPY-positive terminals 1) largely (~75 %) formed symmetric synapses, 2) mainly (~72 %) contained pleomorphic vesicles, 3) commonly targeted small and medium dendrites, and 4) had higher average bouton areas, active zone lengths, and larger vesicle pools as compared to the overall GABAergic ICc population.

## 2. Materials and methods

### 2.1. Animals

All procedures were conducted in accordance with the Northeast Ohio Medical University Institutional Animal Care and Use Committee and NIH guidelines. Results are described from 4 male Fischer Brown Norway (FBN) rats (National Institute of Aging; Bethesda, MD, USA; RRID:SCR_007317). Rats used for this study were aged 2–3 months. Efforts were made to minimize the number of animals and their suffering.

### 2.2. Perfusion and sectioning

Animals were deeply anesthetized with isoflurane and perfused transcardially with Tyrode’s solution, followed by 250 ml of 1 % glutaraldehyde and 4 % paraformaldehyde in 0.1 M phosphate buffer at a pH 7.4. The brain was then removed and stored overnight at 4 °C in 1 % glutaraldehyde and 4 % paraformaldehyde in 0.1 M phosphate buffer. The brain was prepared the next morning by removing the cerebellum and cortex and blocking the remaining tissue with transverse cuts posterior to the cochlear nucleus and anterior to the thalamus. The tissue was then cut into 50 μm thick transverse sections with a Vibratome (VT1000S, Leica Microsystems, Buffalo Grove, IL, USA). The tissue was collected in six series. Series were processed as described below or placed in freezing buffer and stored for future processing.

### 2.3. Tissue processing for EM

**Fig. 1.**
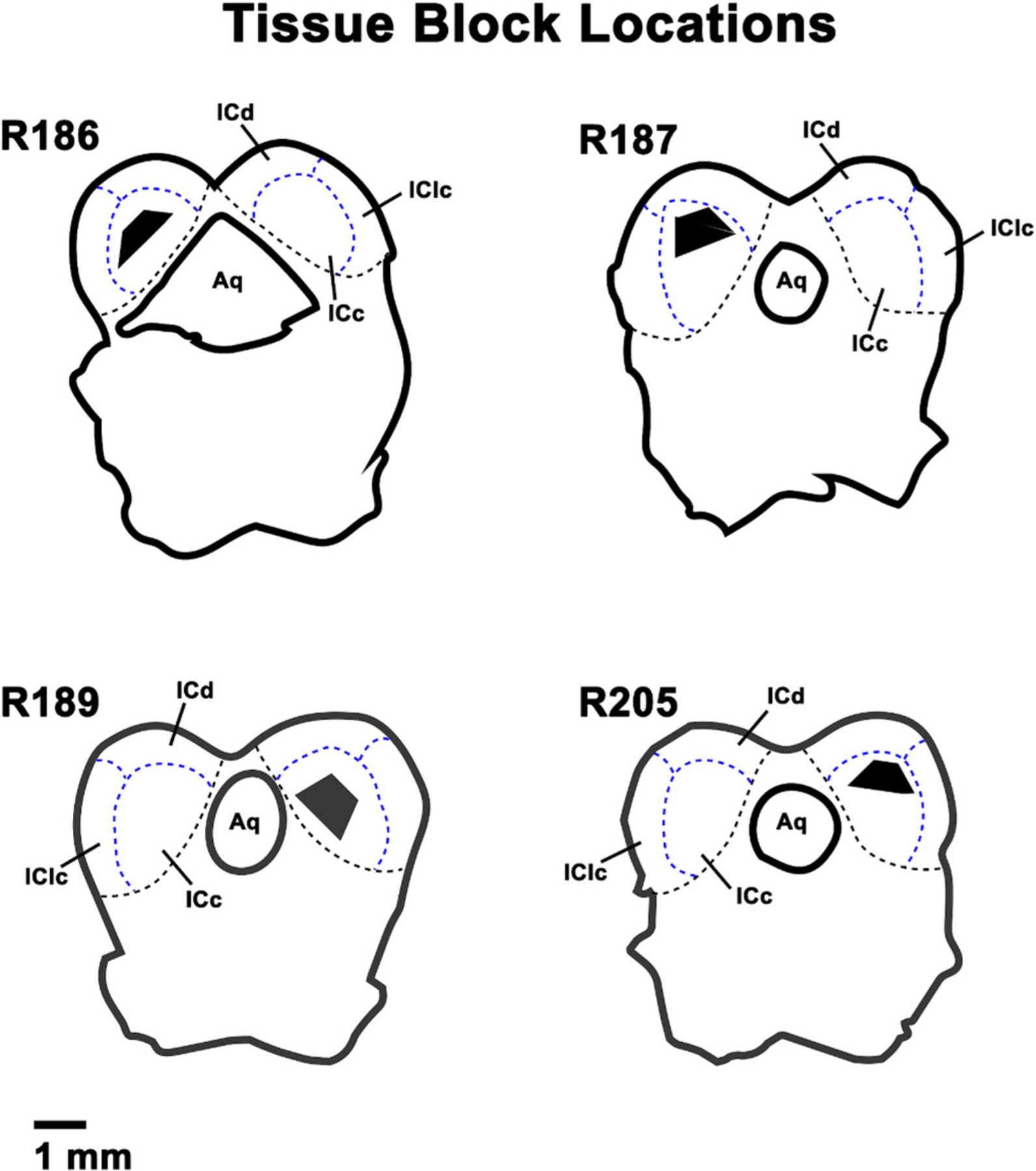
Line drawings of transverse sections through the IC showing the locations of the 4 tissue blocks (black trapezoids) collected for analysis. Dashed lines demonstrate the approximate borders between the IC subdivisions [i.e., central (ICc), dorsal cortex (ICd), or lateral cortex (IClc)]. Each block was confined to the ICc. 4v = 4th ventricle; Aq = Aqueduct.

For one series, sections were processed for immunogold EM following a protocol similar to our previous IC EM studies (Mafi et al., 2022; Mellott et al., 2024). Briefly, the tissue underwent post-fixation in 1 % osmium tetroxide for 30 min, dehydration in a stepwise series of alcohols (50 %, 70 %, 95 %, 100 % and 2× propylene oxide), and embedding in Durcupan resin (Sigma-Aldrich; Millipore Sigma, Burlington, MA, USA). The tissue was then flat-mounted between sheets of Aclar Embedding Film (Ted Pella, Inc., Redding, CA, USA) at 60 degrees Celsius for 48–72 h. Mid-rostrocaudal IC sections (between interaural levels 0.24 mm – 0.72 mm; Paxinos and Watson, 1998) were examined with brightfield stereomicroscopy. Trapezoidal blocks, with a 1 mm base and 0.7–0.8 mm height, were extracted from the ICc (black trapezoids, Fig. 1). Initial borders of the ICc were delineated according to the rat anatomical atlas of the brain (Paxinos and Watson, 1998). Osmium fixation revealed the conspicuous lateral lemniscal fibers that course between the ICc and the IClc. Our libraries of glutamate decarboxylase (GAD) immunoreactivity in EM prepared tissue, adjacent sections stained for Nissl substance or cytochrome oxidase (CO), and our experience with EM in the IC guided our block trimming to ensure samples did not contain tissue from the non-lemniscal IC (Nakamoto et al., 2013; Mellott et al., 2014c; Mafi et al., 2022; Mellott et al., 2024). Once each trapezoidal block was carved out using a razor blade, the tissue was glued to a cylindric resin base with cyanoacrylate (Krazy Glue, Columbus, OH, USA).

Ultrathin sections (50 nm) were taken with an ultramicrotome (UC6 Ultramicrotome, Leica Microsystems, Buffalo Grove, IL, USA). For each tissue block, every twelfth section was collected to ensure a singular synapse was not analyzed twice. Sections were collected onto 200-mesh Formvar coated nickel grids (Electron Microscopy Science, Hatfield, PA, USA). A total of eight grids, each with a single ICc ultrathin section, were collected per case. Briefly, (see: Nakamoto et al., 2013; Mellott et al., 2014c), sections were dried for three hours and were then placed overnight into anti-NPY monoclonal antibody (mouse anti-NPY, Sigma Aldrich, St. Louis, MO; WH0004852M1) diluted 1:500 in 0.05 M Trisbuffered saline with 0.1 % Triton X-100, pH 7.6 (TBST). The next day the sections were washed in TBST pH 7.6, then washed in TBST pH 8.2, and placed into a secondary antibody conjugated to 10 nm gold particles (goat anti-mouse, diluted 1:100 in TBST pH 8.2; Abcam Limited, Cambridge, UK; ab39619). Lastly, sections were washed in TBST pH 7.6, washed in Nanopure water, stained with uranyl acetate (2 % aqueous) and Reynold’s lead citrate (Reynolds, 1963), and airdried.

### 2.4. EM imaging

Four blocks of tissue from male FBN rats with superior ultrastructure were chosen. A 5-point scale was used to grade the intactness and quality of ultrastructure. Only tissue with a score of 4 or 5 was quantified. Our 5point scale reflects a combination of successful fixation, immunogold processing and absence of electron dense artifacts. Scores of 4 & 5 yield clear ultrastructure with easily identifiable NPY-positive profiles that are readily resolved. Ultrastructure was imaged with a transmission electron microscope (JEM-1400Plus, JEOL, Peabody, MA, USA) at an accelerating voltage of 80 kV and at a magnification of 50,000. Based on experience, a magnification of 50,000 ensures that symmetric synapses in the inferior colliculus are discernable. Tissue was digitally imaged with an Orius or Rio9 side mount camera (Gatan, Pleasanton, CA, USA). Images of ultrastructure were taken with Gatan Microscopy Suite Software (GMS3, Gatan, Pleasanton, CA, USA) integrated and calibrated with SerialEM Tomography software (Mastronarde, 2003). SerialEM is a gold standard for analytical applications in biological TEM and facilitated efficient imaging, analysis, and data recording. For each tissue block, we collected photomontages from each of 8 grids for a total image area of 11,300 μm^2^. Adobe Photoshop (Adobe Systems, Inc., San Jose, CA, USA) was used to add scale bars, crop images, adjust intensity levels globally and colorize monochrome images.

### 2.5. Analysis of NPY profiles

NPY synapses were identified by presynaptic terminals that were labeled with immunogold, contained collections of presynaptic vesicles (often pleomorphic), and displayed synaptic junctions (Rockel and Jones, 1973; Jones and Rockel, 1973; Peters et al., 1991; Paloff and Usunoff, 1992; Helfert et al., 1999; Nakamoto et al., 2013; Mafi et al., 2022; Mellott et al., 2024). Synaptic junctions were assigned as symmetric or asymmetric based on the morphology prominence of the postsynaptic density (Peters et al., 1991; Peters and Palay, 1996). As is common with immunostaining protocols, there are variations in background staining from section to section. To address this, assessments of immunolabeling were performed with comparisons to nearby structures (discussed in detail in prior reports; Nakamoto et al., 2013, 2014; Mellott et al., 2014b, Mafi et al., 2022; Mellott et al., 2024). Aggregates of two or more gold particles were quantified as a single particle and gold particles found clumped together on mitochondria or on membranes were left out of quantification as this is indicative of non-specific binding. Profiles with three or more gold particles overlying the cytoplasm were considered NPY-positive (Hoerder-Suabedissen et al., 2018; Glass et al., 2002; Duffy et al., 2011; Omelchenko and Sesack, 2006). Postsynaptic targets were identified as dendrites, spines, boutons, and somata.

### 2.6. Data analysis

We examined 45,200 μm^2^ of ICc tissue across four 2–3 month-old FBN rats (Fig. 1). Each presynaptic NPY-positive terminal and postsynaptic target was analyzed manually with ImageJ (Schneider et al., 2012). We collected several ultrastructural details for each synapse: 1) presynaptic area, 2) active zone length, 3) the total number of vesicles in the profile and 4) the number of vesicles at the active zone (ready releasable pool), and 5) the number and ultrastructural grade of each presynaptic mitochondrion (Coughlin et al., 2015; Smith et al., 2016; Mafi et al., 2022). For the sake of consistency across EM studies in the IC, we classified dendritic sizes by measuring the shortest diameter (>1.5 μm-large; 0.5 to 1.5 μm-medium; <0.5 μm-small) according to Helfert et al. (1999). As detailed in Mafi et al. (2022) it may be difficult to distinguish spines from small dendrites in the IC as 1) there is overlap in diameter ranges, 2) spine necks are not consistently observed at 50 nm thick sections, 3) spine apparatus is only present in a subset of spines, and 4) cytoskeleton is not always readily defined in such small profiles. Additionally, the lower glutaraldehyde fixation often made it more difficult to identify characteristics of smaller profiles. Thus, it is quite possible that we inappropriately characterized a postsynaptic spine as a postsynaptic small dendrite.

Variation in synaptic junction symmetry, presynaptic area, the active zone length, number of vesicles, presynaptic mitochondria and postsynaptic targets, were analyzed using linear mixed-effects models. Mixed-effects models allow for a hybrid of repeated measures analysis (i.e., “within-subject” variables), Model I ANOVA fixed factor analysis (i.e., “between-subject” variables), and Model II ANOVA random factor analysis (i.e., variance components), in the same gestalt statistical test. In this study, ultrastructural profile was specified as a fixed factor within individual rats, and individual rat number was specified as a random factor. Models were fit assuming a gaussian error distribution (R function lmer). Given low sample sizes, all variables were rank-transformed prior to model fitting, such that statistical tests were equivalent to non-parametric tests (Conover and Iman, 1981). The accuracy of all model fits was assessed by visual inspection of the distribution of residuals versus fitted values and examination of the dispersion of standardized residuals (to evaluate the homogeneity of group variances (using the “check_model” function of the “performance” package in R). All statistical tests were performed in R (version 4.4.3 for Mac OS X; R Core Team, 2025), supplemented by the add-on packages lme4 (Bates et al., 2015), lmerTest (Kuznetsova et al., 2009), emmeans (Lenth, 2024), performance (Lüdecke et al., 2021), and tidyverse (Wickham et al., 2009).

To better understand where NPY neurons fit within the broader GABAergic population, we compared data from the current study to data from our previous study on the GABAergic neuronal population in the ICc (Wawrzyniak et al., 2025). To facilitate direct comparisons between the two data sets, we log_10_-transformed continuous bouton area, active zone length, and total vesicle pool measurements from the total agematched population of GABAergic cells across the ICc in our previous study (Wawrzyniak et al., 2025). The log transformed GABAergic bouton areas were binned into tercile categories (“small” [<0.539 μm^2^], “medium” [0.539–0.824 μm^2^], and “large” [>0.824 μm^2^] bouton areas) based on the distribution of the GABAergic bouton population. These values are consistent with the generally accepted presynaptic areas of <0.6 μm^2^ as “small” and > 1.0 μm^2^ as “large.” Log transformed GABAergic active zone length were binned into terciles (“small” [<177.5 nm], “medium” [177.5–259 nm], and “large” [>259 nm] terminal zone lengths) based on the distribution of the GABAergic active zone population. Log transformed GABAergic total vesicle pools were binned into terciles (“small” [<43 vesicles], “medium” [43–67 vesicles], and “large” [>67 vesicles] pools) based on the distribution of the GABAergic vesicle population. Log-transformed symmetric NPY-positive bouton areas, active zone lengths, and vesicle pools were then compared to these categories using chi-squared tests. This approach was chosen to provide a summary-level visualization of the morphological distribution shifts. This categorical comparison serves as an illustrative supplement to our findings in the current study and the findings from our prior study (Wawrzyniak et al., 2025).

## 3. Results

We examined the ultrastructure of NPY-positive synapses across 45,200 μm^2^ of ICc of adult (2–3 month) FBN rats. We quantified 68 NPY-positive synapses. We first describe the general morphology of NPY-positive synapses in the ICc. We then provide descriptions of post-synaptic targets (dendrites, spines, boutons, and somata). We then present data regarding the presynaptic elements, including terminal size, gold density, post-synaptic target size, active zone length, vesicle pools, and mitochondria as well as synaptic junction symmetry. Finally, we compare symmetric NPY-positive presynaptic elements from this study to GABAergic presynaptic elements from our previous study (Wawrzyniak et al., 2025).

### 3.1. General morphology of NPY-positive synapses in the ICc

**Fig. 2.**
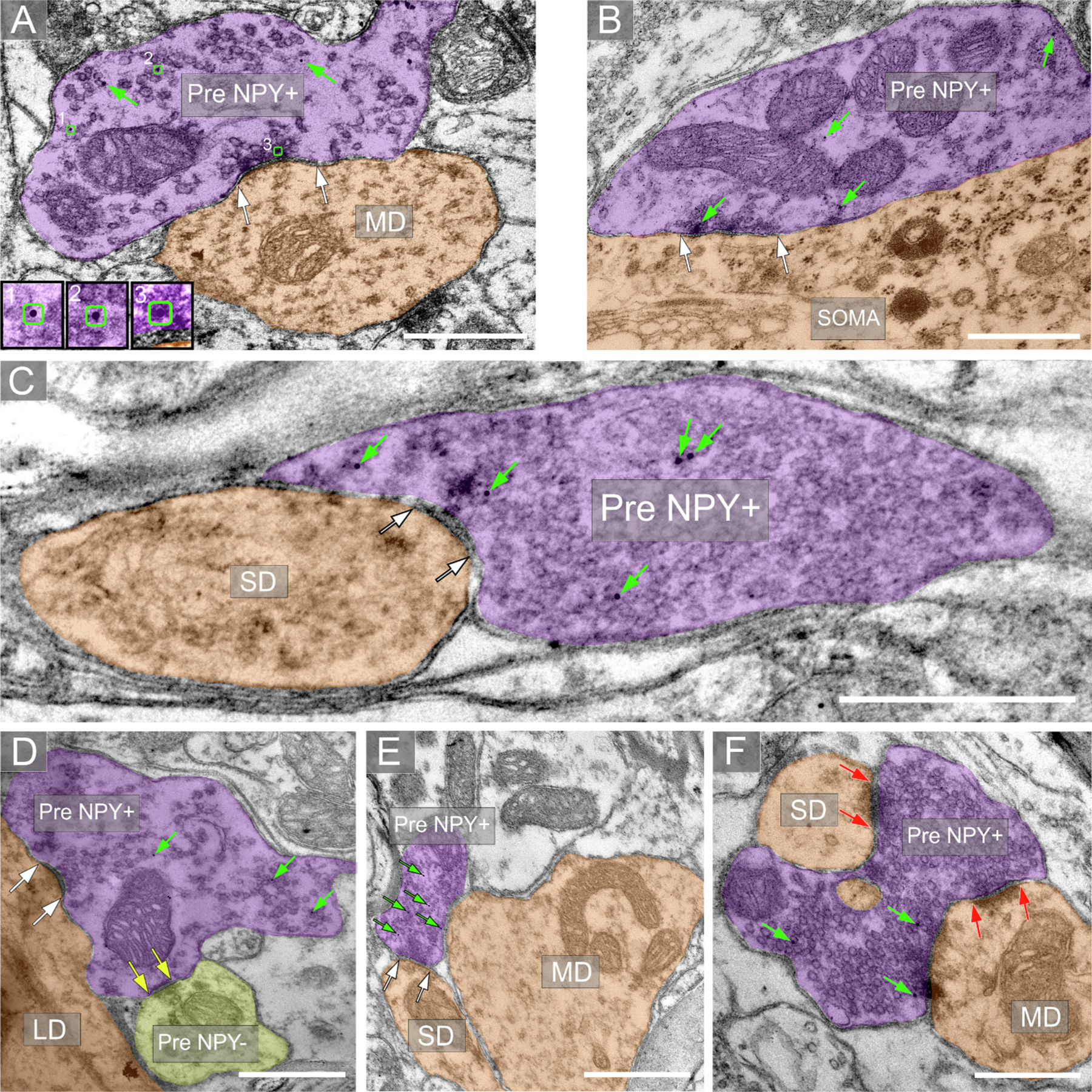
Electron micrographs showing NPY-positive synaptic ultrastructure in the ICc. NPY-positive (Pre NPY+) terminals are pseudo-colored purple. Black dots demonstrate immunogold labeling of NPY, designated by green arrows (A-F) or green boxes (A). NPY-negative (Pre NPY-) terminals are pseudo-colored yellow. NPY-negative postsynaptic (Post NPY-) targets are pseudo-colored orange. Symmetric NPY-positive synapses are indicated by pairs of white arrows. Asymmetric NPY-positive synapses are indicated by pairs of red arrows. A) electron micrograph of a large (>0.824 μm^2^) NPY-positive presynaptic bouton forming a symmetric synapse onto a medium dendrite. Insets 1–3 demonstrate enlarged views of the boxed immunogold particles. B) electron micrograph of a large (>0.824 μm^2^) NPY-positive presynaptic bouton forming a symmetric synapse onto a soma. C) electron micrograph of a medium (0.539–0.824 μm^2^) NPY-positive presynaptic bouton forming a symmetric synapse onto a small dendrite. Image enlarged to demonstrate synaptic elements, pleomorphic vesicles, and immunogold particles positive for NPY. D) electron micrograph of a large (>0.824 μm^2^) NPY-positive presynaptic bouton forming a symmetric synapse onto a large dendrite and an NPY-negative presynaptic terminal forming an axo-axonic synapse onto the NPY-positive bouton (yellow arrows). E) electron micrograph of a small (<0.539 μm^2^) NPY-positive presynaptic bouton forming a symmetric synapse onto a small dendrite. F) electron micrograph of a large (>0.824 μm^2^) NPY-positive presynaptic bouton with round clear vesicles forming three asymmetric synapses onto a small and medium dendrite. LD – Large Dendrite; MD – Medium Dendrite; SD – Small Dendrite. Scale bars = 500 nm.

Presynaptic NPY-positive terminals forming synapses were identified by the presence of immunogold labeling of NPY (Fig. 2, green arrows). The presence of pre- and post-synaptic densities, a synaptic cleft, and collections of vesicles at the synapse and throughout the presynaptic profile were examined. NPY-positive terminals largely (~75 %) formed symmetric synapses and contained pleomorphic vesicles (Fig. 2A–E, white arrows; Table 1). A percent (~25 %) of the NPY-positive terminals formed asymmetric synapses and contained round vesicles (Fig. 2F, red arrows; Table 2). Axo-axonic contacts onto NPY-positive terminals were also observed (Fig. 2D, yellow arrows).

### 3.2. NPY-positive synapses target small and medium dendrites

**Fig. 3.**
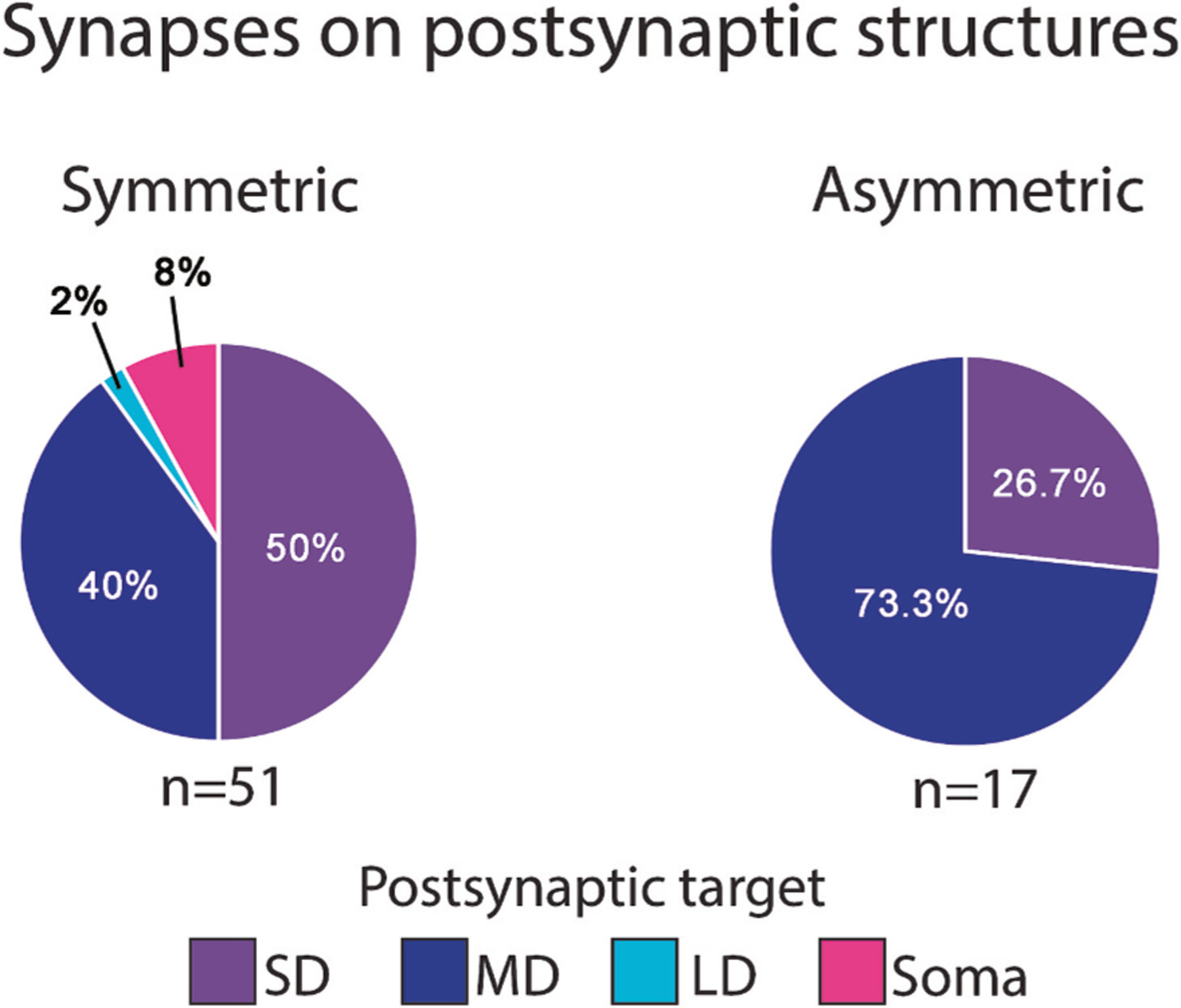
NPY-positive synapses onto postsynaptic targets in the ICc. Pie charts showing the frequency of symmetric and asymmetric NPY-positive terminals forming synapses onto postsynaptic targets. Left pie chart displays post-synaptic targeting frequency for symmetric NPY synapses. Right pie chart displays post-synaptic targeting frequency for asymmetric NPY synapses. Data is presented across four cases. LD – Large Dendrite; MD – Medium Dendrite; SD – Small Dendrite.

We recorded the postsynaptic targets for each NPY-positive synapse (Fig. 3). The postsynaptic profile targets of NPY-positive synapses included small, medium, and large dendrites (SD, MD, LD) and somas (Fig. 2&3). As described in the methods, dendrites were categorized by their diameters as small (<0.5 μm), medium (0.5–1.5 μm), and large (>1.5 μm). Regardless of synapse symmetry, the most common post-synaptic targets were small and medium dendrites (Fig. 3). Symmetric NPY-positive synapses were also found to target large dendrites and somas (Fig. 2&3). We did not find any examples of asymmetric NPY-positive synapses targeting a large dendrite or soma.

### 3.3. NPY-positive boutons, vesicle pools, and mitochondria

**Fig. 4.**
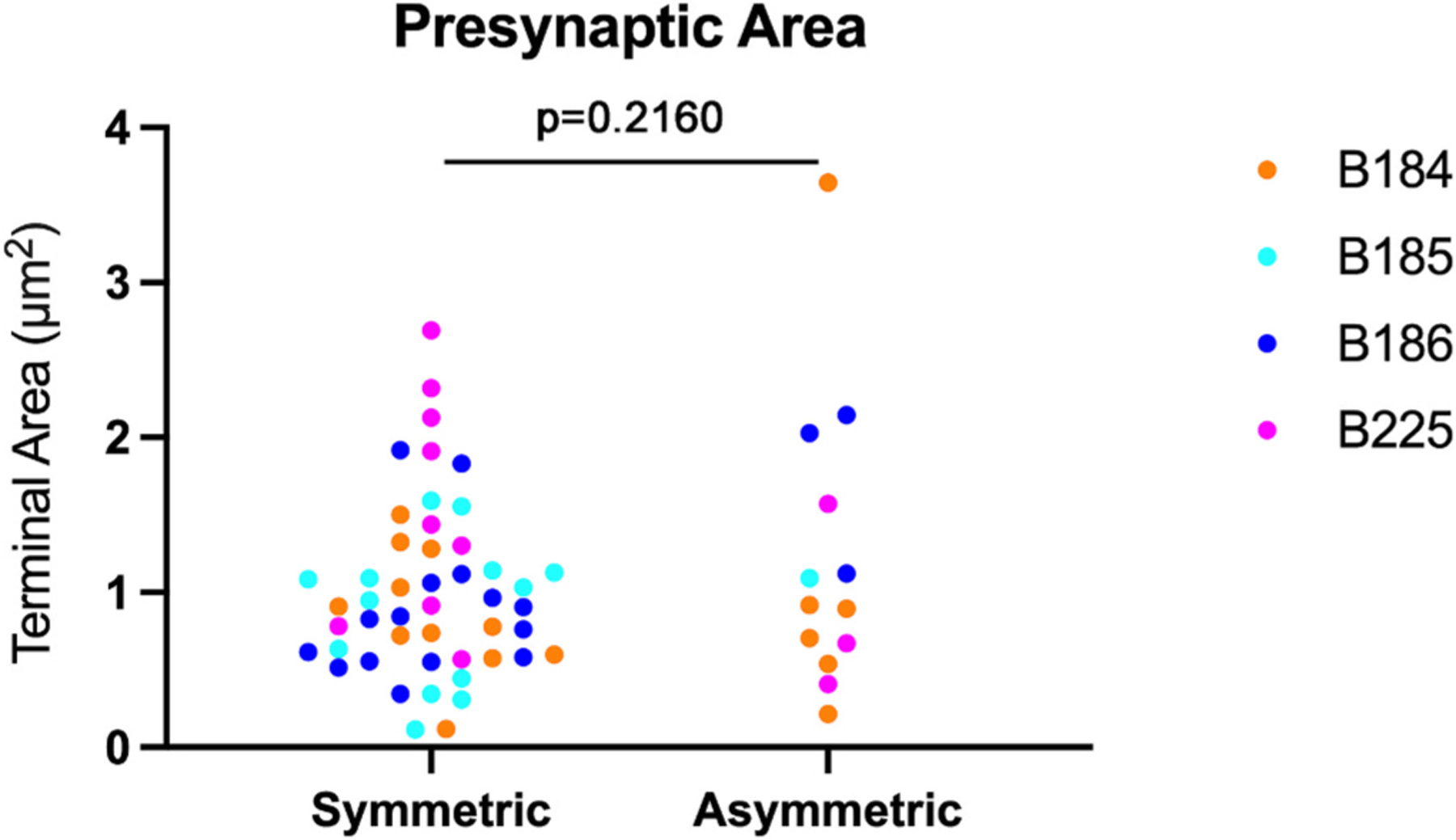
NPY bouton areas in the ICc. Dot plot illustrating the presynaptic bouton areas of symmetric and asymmetric NPY synapses. Small markers indicate individual measurements, with different colors representing different animals. Pairwise differences demonstrated that there was no significant difference between the symmetric and asymmetric groups.

We measured the average size of each symmetric and asymmetric NPY-positive terminal. NPY-positive presynaptic terminals forming symmetric synapses ranged from 0.116 μm^2^ to 2.691 μm^2^, with an average terminal area of 1.01 μm^2^ (Table 1, Fig. 4). NPY-positive pre-synaptic terminals forming asymmetric synapses ranged from 0.214 μm^2^ to 3.646 μm^2^, with an average terminal area of 1.2 μm^2^ (Table 2, Fig. 4). The difference between symmetric and asymmetric-synapse-forming terminal areas was not statistically significant (*p* = 0.216; Fig. 4).

We quantified the number of vesicles in each NPY-positive bouton. Boutons forming symmetric NPY synapses had an average of 82.9 vesicles (Table 1). For the symmetric group, terminal areas and total vesicle pools were significantly positively correlated (ρ = 0.53, *p* < 0.05; Table 3). Boutons that formed asymmetric NPY synapses had an average of 111.5 vesicles (Table 2). In the asymmetric group, terminal areas and vesicle pools were positively correlated (ρ = 0.55, *p* = 0.05; Table 3). As mentioned above, most NPY positive vesicle pools were classified as pleomorphic, meaning they had a mixture of several vesicle shapes (Fig. 2A–E). As opposed to the few pools that had a clear population of largely round vesicles (Fig. 2F). The packing of these vesicles ranged from sparse (Fig. 2A, B, D) to dense (Fig. 2C,F).

**Fig. 5.**
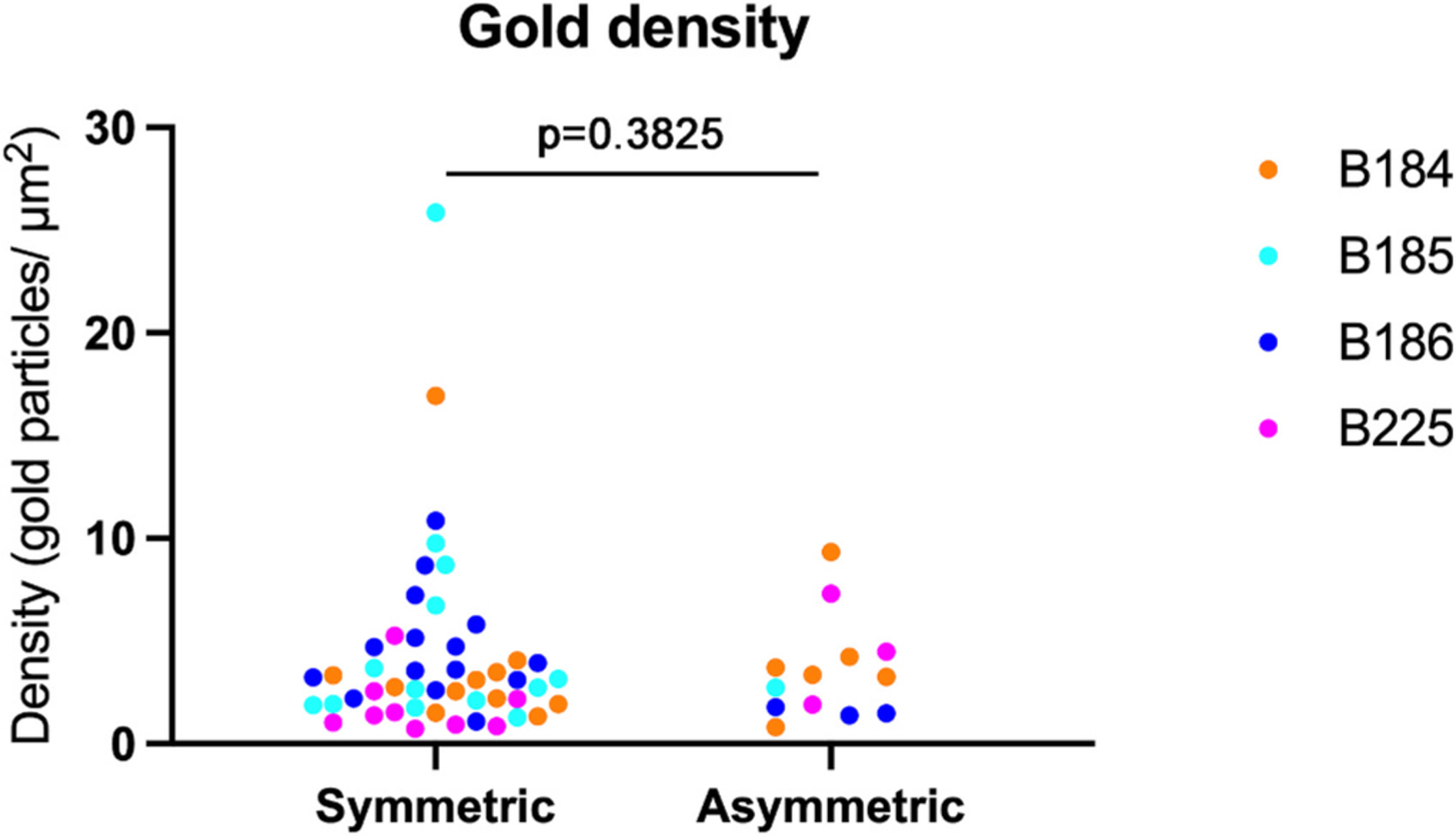
NPY immunogold density. Dot plot illustrating the density of gold particles in NPY-positive boutons. Small markers indicate individual measurements, with different colors representing different animals. Pairwise differences demonstrated that there was no significant difference between the symmetric and asymmetric groups.

To calculate the density of gold particles per bouton area, we divided the number of NPY immunogold particles in each measured bouton by the bouton profile area. The gold density (gold particles/μm^2^) was not significantly different between symmetric and asymmetric NPY groups (Fig. 5). Interestingly, the gold density (gold particles/ μm^2^) was significantly negatively correlated with bouton area for the symmetric NPY synapse group (ρ = −0.86, p < 0.05; Table 3). The same is true for the asymmetric NPY synapse group (ρ = −0.98, p < 0.05; Table 3).

The average number of mitochondria per bouton was 2 for the symmetric NPY group and 2.6 for the asymmetric NPY group (Tables 1 & 2). There was no statistically significant difference between number of mitochondria in symmetric versus asymmetric groups (*p* = 0.1088). Interestingly, the number of mitochondria was significantly positively correlated with bouton area for the symmetric NPY group (ρ = 0.54, p < 0.05; Table 3). For the asymmetric NPY group, the number of mitochondria per bouton was positively correlated (ρ = 0.48, *p* = 0.095; Table 3).

### 3.4. NPY-positive active zone lengths and vesicles at synapse

**Fig. 6.**
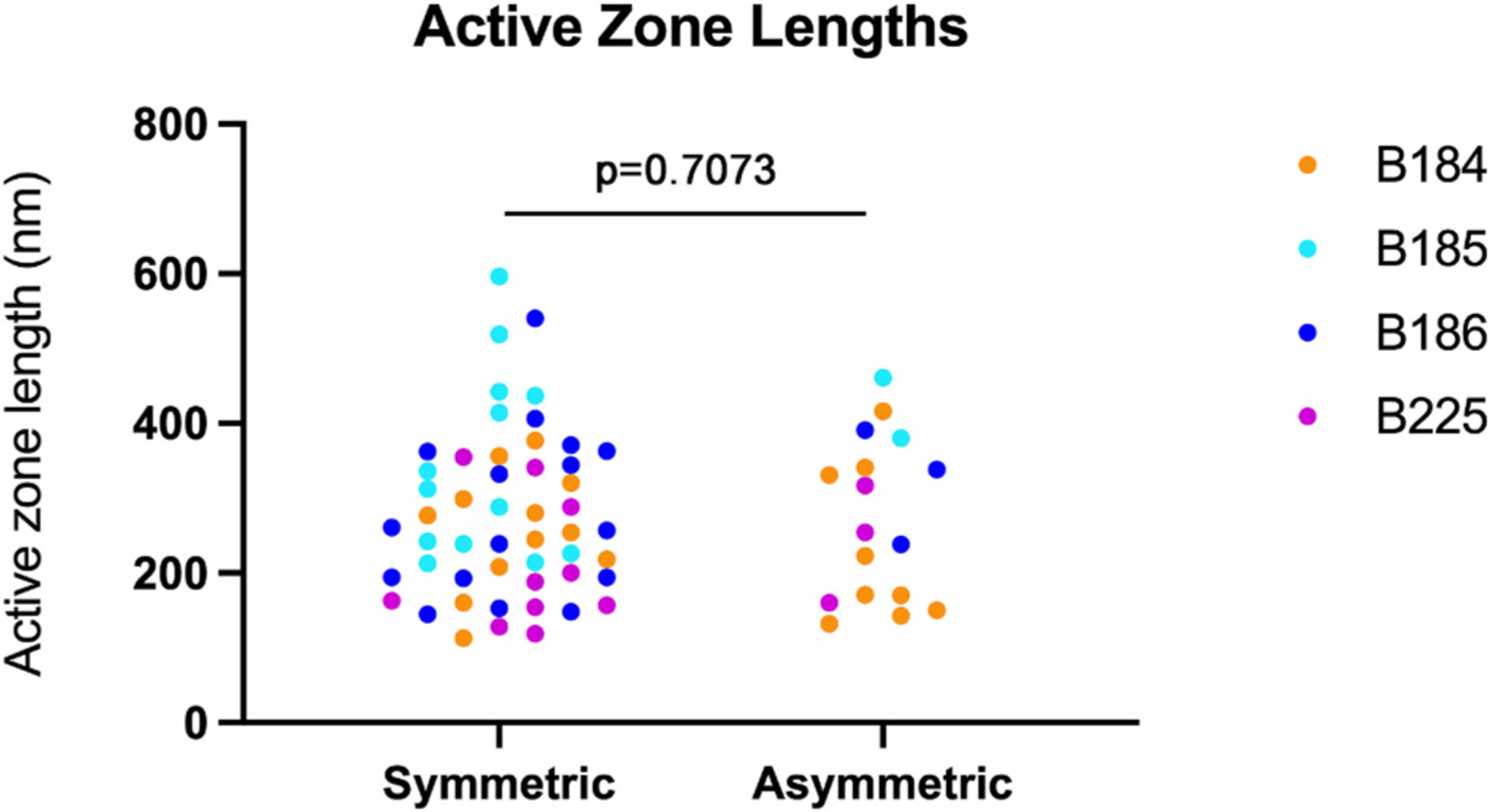
NPY synaptic lengths. Dot plots illustrating the terminal zone lengths of symmetric and asymmetric NPY synapses. Small markers indicate individual measurements, with different colors representing different animals. Pairwise differences demonstrated that there was no significant difference between the symmetric and asymmetric groups.

We measured the length of each NPY-positive active zone and quantified the number of vesicles clustered at the active zone of each presynaptic terminal. Terminals forming symmetric NPY synapses had active zone lengths ranging from 113 nm to 596 nm, with an average of 278 nm (Table 1, Fig. 6). Symmetric terminals had an average of 9.8 vesicles at the active zone (Table 1). Terminals forming asymmetric NPY synapses had active zone lengths ranging from 132 nm to 461 nm, with an average of 271.5 nm (Table 2, Fig. 6). Asymmetric terminals had an average of 10.2 vesicles at the active zone (Table 2). The difference between symmetric and asymmetric active zone lengths was not statistically significant (*p* = 0.7073; Fig. 6). Regardless of synapse symmetry, active zone lengths and number of vesicles at active zone were significantly positively correlated (symmetric: ρ = 0.59, p < 0.05; asymmetric: ρ = 0.68, p < 0.05; Table 3).

### 3.5. GABA-positive and NPY-positive ultrastructural comparisons in the ICc

**Fig. 7.**
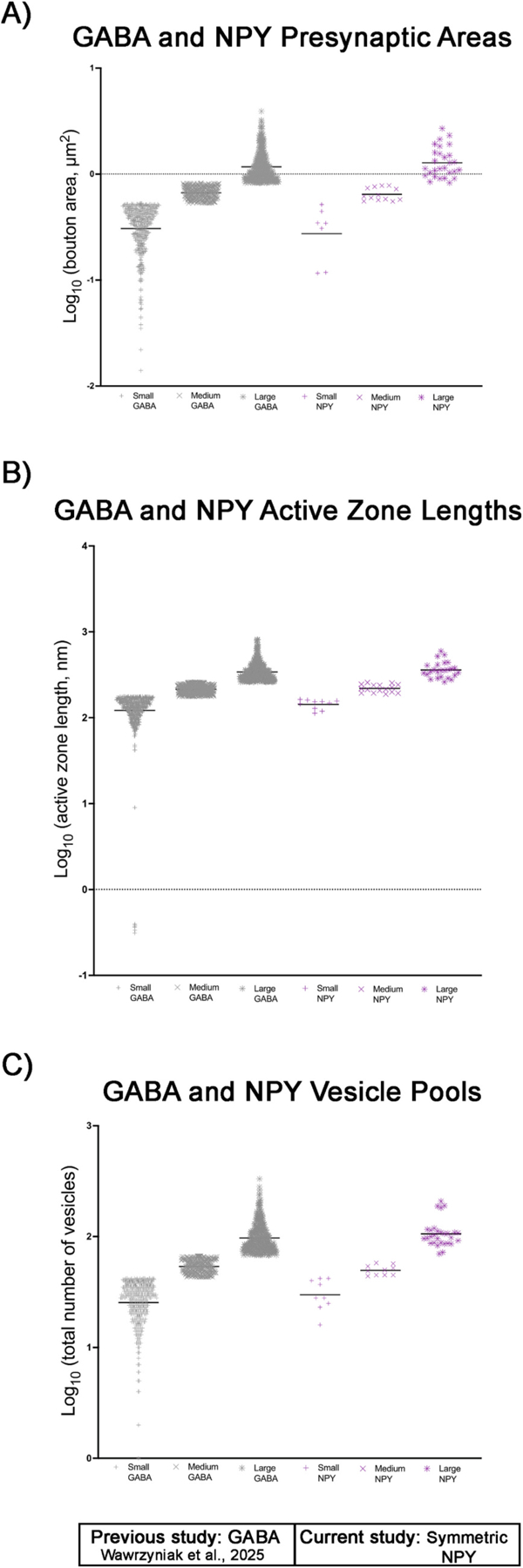
Summary comparisons of size category distributions for presynaptic area, terminal zone length, and vesicle pool size between GABA-positive and NPY-positive boutons in the ICc. Presynaptic areas, terminal zone lengths, and vesicle pools for NPY neurons (purple) in this study compared with GABAergic neurons (gray) from our previous study. Presynaptic areas, terminal zone lengths, and vesicle pools were log_10_-transformed and classified into terciles based on the distribution of the entire ICc GABAergic population. Individual data points were binned into three size categories (small, medium, large). Values in the “small” category represented by (+) symbol, “medium” category represented by the (x) symbol, and “large” category represented by the (*) symbol. Individual data points plotted for each group. Chi-squared tests revealed a significant difference in the distribution of NPY boutons, terminal zone lengths, and vesicle pools, demonstrating that the NPY-positive structures were significantly more likely to fall into the “large” categories (***p < 0.001, presynaptic areas; (*p < 0.05, active zone lengths; ***p < 0.001, vesicle pools). In each dot plot, dark lines represent means of the distribution.

NPY is expressed by a subset of GABAergic cells. We were interested in whether the NPY boutons represent a random subset of the broad GABAergic population or represent a subset that differs in synaptic morphology. We developed a classification scheme from our previous study on GABAergic ICc neurons (Wawrzyniak et al., 2025). We classified GABAergic bouton areas, active zone lengths, and vesicle pools in “small,” “medium,” and “large” size categories by binning the morphological parameters into terciles (see methods). Fig. 7 compares the values for the overall set of GABA synapses (gray, from previous study) and the values for the symmetric NPY synapses (current study, in purple). Chi-squared tests were then used to compare the distributions between GABA-positive and NPY-positive bouton areas, active zone lengths, and vesicle pools. Statistical analysis showed that the NPY-positive presynaptic areas were significantly more likely to fall into the “large” category than expected based on the distribution of GABAergic presynaptic areas (***, *p* < 0.001; Fig. 7A). Similarly, NPY-positive active zone lengths were significantly more likely to fall into the “large” category (*, p < 0.05; Fig. 7B). Not surprisingly, NPY-positive vesicle pools were significantly more likely to fall into the “large” category (***, p < 0.001; Fig. 7C).

## 4. Discussion

The present study describes the ultrastructure and synaptic organization of NPY-positive synapses and compares them to the overall GABAergic neuronal population across the ICc. The majority (~75 %) of NPY-positive terminals formed symmetric synapses and ~ 72 % contained pleomorphic vesicles, which is consistent with inhibitory neurotransmission. Less commonly (~25 %) we observed NPY-positive boutons with asymmetric synapses and round vesicles, which is suggestive of excitatory neurotransmission. We outline several tentative explanations for this surprising finding below. We quantified NPY terminal areas, gold density, active zone lengths, and mitochondria and found no significant differences between the symmetric and asymmetric groups. We ran correlations between the ultrastructural criteria that we quantified and found that most correlation patterns were consistent between the symmetric and asymmetric groups. Finally, to contextualize our findings within the broader GABAergic ICc population and determine whether NPY boutons represent a random subset of the GABAergic population or a subset that differs in synaptic morphology, we compared the present symmetric NPY synapse dataset to the distribution of the GABAergic population from our previous study. Notably, NPY-positive presynaptic areas showed a significantly different distribution, with a greater proportion of NPY boutons falling into the “large” size category. A similar pattern was observed for active zone lengths and vesicle pools, reinforcing the conclusion that NPY boutons exhibit distinct morphological characteristics. Large boutons are often associated with greater postsynaptic effects, which highlights the likelihood that NPY neurons play a role in regulating excitation. We discuss the relationship of the current report to previous studies on GABAergic synapses in the ICc. Separately, we comment on the surprising finding of NPY-positive terminals with morphology suggestive of excitation. Lastly, we present a brief consideration of technical issues related to the current study before concluding with the functional implications.

### 4.1. Comparisons to previous studies

The current report sought to characterize NPY synaptic ultrastructure to expand upon our previous work on GABAergic synaptic ultrastructure of FBN rats across the tonotopic ICc (Wawrzyniak et al., 2025). In the prior study, we established that at young age (3–4 months), the density of GABAergic synapses and their morphological characteristics did not differ along the tonotopic axis of the ICc (Wawrzyniak et al., 2025). This observation allowed us to extract larger tissue samples likely associated with a wide range of frequencies. GABAergic presynaptic areas ranged from 0.014 μm^2^ to 3.91 μm^2^, which encompasses the NPY bouton ranges, 0.116 μm^2^ to 3.646 μm^2^, from the current study. NPY bouton areas were larger, with an average area of 1.1 μm^2^, whereas the average GABAergic bouton area was 0.75 μm^2^. In line with these findings, we discovered that active zone lengths and the number of vesicles clustered at active zones were larger in NPY boutons (average active zone length, 276.4 nm; average number of vesicles at synapse, 9.9 vesicles). The average GABAergic active zone length was 232.3 nm and the average number of vesicles at synapse was 8.3 vesicles. Likewise, NPY boutons tended to contain large vesicle pools and a greater number of mitochondria per bouton, with an average of 89 vesicles and 2.1 mitochondria per bouton. In contrast, GABAergic boutons had an average of 60.9 vesicles and 1.5 mitochondria per bouton. A linear relationship between bouton size and its morphological characteristics (active zone length, vesicle number, mitochondrial volume) is consistent throughout many brain regions (Pierce and Lewin, 1994; Yeow and Peterson, 1991; Streichert and Sargent, 1989; Peters and Harriman, 1990). Interestingly, the larger size of NPY boutons implies that NPY synapses may have greater postsynaptic effects than non-NPY-expressing GABAergic synapses. This interpretation aligns with previous studies on NPY in the IC, which have shown that NPY is co-expressed by ~30–40 % of GABAergic cells in the IC, whereas the NPY Y1 receptor is co-expressed by ~80 % of glutamatergic neurons in the IC, enabling NPY to have extensive neuromodulatory effects in the IC (Silveira et al., 2020, 2023; Almassri et al., 2025). The current study also demonstrated that NPY inputs preferentially target small and medium dendrites. Collectively, NPY is positioned to be a major neuromodulator of neuronal excitation in the IC.

### 4.2. Excitatory NPY-positive boutons

In the IC, it is generally well accepted that synapses with symmetric junctions support inhibitory neurotransmission, while synapses with asymmetric junctions support excitation (Helfert et al., 1999; Ito et al., 2009; Nakamoto et al., 2013, 2014; Mafi et al., 2022; Mellott et al., 2024; Wawrzyniak et al., 2025). However, perhaps the most exciting element of our data, in contrast to the canonical view that NPY is primarily co-expressed by inhibitory GABAergic neurons across brain regions (Polgár et al., 2011; Ramamoorthy et al., 2011; Silveira et al., 2020; Almassri et al., 2025), our data reveals that a sizable (~25 %) proportion of morphologically excitatory boutons contain NPY-immunoreactive gold particles. These boutons exhibit classic excitatory features, including round vesicles and asymmetric synapses, suggesting that NPY may be present in a handful of glutamatergic terminals in the auditory midbrain. Based on the prior work on GABAergic synapses, it is unclear why NPY would be found in these terminals forming asymmetric synapses, however we will speculate on a few potential explanations. First, the relationship between inhibitory neurotransmitter, the vesicle shape, and asymmetry of the PSD does not always fall perfectly in line. Helfert et al. (1999) reported a small percentage of GABAergic terminals with round vesicles and conversely reported a small percentage of GABA-negative terminals with pleomorphic shaped vesicles. In our previous EM studies, our unpublished observation is that ~1 % of GABA-labeled boutons in the 2–4-month-old FBN rat display excitatory synaptic characteristics. Perhaps most interestingly, Liu et al. (1999) examined glycinergic ultrastructure in the cat IC and concluded that 25 % of the glycine-positive terminals formed asymmetric PSDs. Additionally, ultrastructural characterization of NPY in the rat hippocampus revealed findings strikingly similar to the current study, where despite the abundance of symmetric NPY synapses, they also observed NPY-labeled terminals forming asymmetric synapses (Milner and Veznedaroglu, 1992).

A second consideration is the unique internalization and recycling mechanisms of NPY receptors. When NPY is bound to its postsynaptic NPY Y1 receptor on a glutamatergic cell surface, there can be a rapid NPY-induced internalization and recycling of the NPY Y1 receptor complex, which has been shown in other brain regions (Kempf et al., 2015; Holliday et al., 2005; Gicquiaux et al., 2002). Such internalization could allow for NPY peptide expression in excitatory neurons. On the other hand, the presynaptic NPY Y2 receptor has been shown to undergo a similar internalization mechanism, but at a slower rate and to a lesser degree (Parker et al., 2008; Lundell et al., 2011). To our knowledge, the presynaptic NPY Y2 receptor has not been studied in the IC. Future studies are needed to determine whether these NPY internalization and recycling mechanisms occur in the IC and what functional roles they serve.

Lastly, perhaps NPY is indeed synthesized in a small percentage of glutamatergic cells. In conversations with colleagues, it was noted that RNA-sequencing data of NPY in the IC consistently shows a small, often negligible, percentage of NPY co-expression by glutamatergic cells (unpublished findings, Michael Roberts). Ultimately, it will be worthwhile to determine the strictness with which one can apply the morphology of a synapse to a respective function based on a given neurotransmitter.

### 4.3. Technical considerations

We employ immunogold-EM to detect NPY synaptic profiles in male adult (2–3 months) FBN rats. To achieve our findings, several technical considerations had to be navigated. Taking into consideration that this is the first EM study in the IC to examine NPY ultrastructure, we employed various evaluation methods to choose an antibody and concentration that yielded the best results. We tested five different anti-NPY antibodies from recommended sources (polyclonal rabbit anti-NPY, antibody ID # N3266, ImmunoStar, Hudson, Wisconsin; polyclonal rabbit anti-NPY, antibody ID # T-4070, BMA Biomedicals, Switzerland; polyclonal rabbit anti-NPY, antibody ID # ab30914, Abcam Limited, Cambridge, UK; polyclonal rabbit anti-NPY, antibody ID # N9528, Sigma Aldrich, St. Louis, MO; monoclonal mouse anti-NPY, antibody ID # WH0004852M1, Sigma Aldrich, St. Louis, MO). We ran immunohistochemistry for each antibody on our FBN rat tissue, as well as CBA/Caj and NPY-hrGFP mice, courtesy of Dr. Alexander Galazyuk’s and Dr. Michael Roberts’ labs, respectively. We also ran standard controls, including primary and secondary antibody omissions. Western blots were provided by the manufacturers. Two anti-NPY antibodies which showed superior labeling at the fluorescent level, were then taken to the EM level. We tested antibody concentrations ranging from 1:250 to 1:5000 using our pre-embedding 3,3′-diaminobenzidine (DAB) EM and post-embedding immunogold EM protocols (see Mafi et al., 2022; Mellott et al., 2024). The concentration of 1:500 yielded the highest level of DAB and immunogold co-localization. DAB staining tends to fill the boutons and obscure visualization of vesicle shape so we proceeded with post-embedding immunogold for the current study.

To identify NPY ultrastructure with immunogold techniques, we ran several control series to determine what level of glutaraldehyde fixation was necessary to obtain positive label against the background. Ultimately, we used 1 % glutaraldehyde because higher concentrations (such as 2–2.5 % typically used for GABAergic EM) eliminated gold labeling with the NPY antibodies. A lower glutaraldehyde percentage (e.g., 0.5 %) also yielded positive NPY label, but did not result in denser gold label or denser populations of terminals. Thus, we chose to proceed with 1 % glutaraldehyde as it results in better ultrastructural details than 0.5 %. Although pleomorphic and flat vesicle shapes are likely an artifact due to fixation, studies have demonstrated that presynaptic terminals containing vesicles of different shapes and sizes, pleomorphic, are commonly inhibitory (Peters et al., 1991; Peters and Palay, 1996). Given that we used a reduced level of glutaraldehyde, it is difficult to assume that vesicle shape in the current study may or may not provide as a strong metric to determine the inhibitory or excitatory nature presynaptic terminals that contain NPY. These issues, along with the role of the “excitatory NPY-positive boutons” will hopefully be worked out with our future studies.

### 4.4. Functional implications

Throughout the brain, NPY is involved in several mechanisms important for neurological health maintenance, including neuroprotection, calcium homeostasis, protection against oxidative stress and mitochondrial dysfunction, dampening of excitotoxicity, and reduction of neuroinflammation (see reviews: Botelho and Cavadas, 2015; Li et al., 2020; Chen et al., 2019; Duarte-Neves et al., 2016). These effects are of particular interest when looking at the aging brain and age-related disease states. Interestingly, NPY mediates the activation of protein kinase A and p38K, resulting in a downstream effect of glutamate-receptor-overactivity inhibition and rescue of hippocampal, cortical, and retinal cells from necrosis and apoptosis (see review: Li et al., 2020). In the auditory midbrain, NPY signaling dampens recurrent excitation via the Y1 receptor on glutamatergic neurons (Silveira et al., 2023). Additionally, our previous study on NPY mRNA in the aging auditory midbrain demonstrated that NPY mRNA expression remains intact as the brain ages (Almassri et al., 2025). This is in contrast to the overall GABAergic population, which is downregulated in the aging IC (Wenstrup, 2005; Caspary et al., 1990, 1995, 2008; Syka, 2020; Burianova et al., 2009; Gutíerrez et al., 1994; Raza et al., 1994; Ouda and Syka, 2012; Rabang et al., 2012; Milbrandt et al., 1994, 1996, 1997; Pal et al., 2019). This GABAergic downregulation has been linked to the central neural gain hypothesis, where reduced excitation from the periphery is coupled with a reduction of GABAergic neurotransmission centrally (Ono and Oliver, 2014; Ibrahim and Llano, 2019; Knipper et al., 2022; Rumschlag et al., 2022; Ouda et al., 2015). GABA continues to decline into old age, leading to an imbalance of excitation and inhibition, and resulting in dysfunctional temporal coding, signal-to-noise ratios, and shaping of acoustic signals (Palombi and Caspary, 1996a, 1996b; Walton et al., 1998, 2002; Walton, 2010; Frisina, 2001; Frisina and Rajan, 2005; Frisina, 2009; Šuta et al., 2011; Parthasarathy and Bartlett, 2011; Pollak et al., 2011; Cai et al., 2014; Ono and Oliver, 2014; Ibrahim and Llano, 2019; Knipper et al., 2022; Rumschlag et al., 2022; Ouda et al., 2015). Thus, it appears that aging may be preferentially affecting GABA neurons that do not express NPY, while neurons co-expressing GABA and NPY may be playing a critical role in maintaining function in the auditory midbrain. Our lab has ongoing studies exploring the age-related differences in NPY synapses across the IC. Given the complexity and potential of NPY signaling, future studies are needed to determine the role of NPY and its receptor family in the central auditory system.

## Figures and Tables

**Table 1:** Symmetric NPY+ characteristics.

Animal ID	Area Examined (μm^2^)	# NPY synapses	Bouton Area (μm^2^)	Synaptic length (nm)	Ave. # mito	Vesicles (at synapse)	Vesicles (total)
B184 (R186) 2–3 mo	11,300	12	0.87	258.9	1.3	7.4	87
B185 (R187) 2–3 mo	11,300	13	0.88	344.5	2	13.1	76.1
B186 (R189) 2–3 mo	11,300	16	0.89	281.4	1.6	10.6	74.9
B225 (R204) 2–3 mo	11,300	10	1.56	209.3	3.3	7.3	100.9
**Totals/Averages**	**45,200**	**51**	**1.01**	**278**	**2**	**9.8**	**82.9**

Summary of the ultrastructural characteristics of symmetric NPY synapses across the ICc. Symmetric synapses made up a majority of the NPY+ synapses in the ICc. As expected, these synapses mainly contained pleomorphic vesicles.

**Table 2:** Asymmetric NPY+ characteristics.

Animal ID	Area Examined (μm^2^)	# NPY synapses	Bouton Area (μm^2^)	Synaptic length (nm)	Ave. # mito	Vesicles (at synapse)	Vesicles (total)
B184 (R186) 2–3 mo	11,300	9	1.2	230.8	2.3	8.6	142.8
B185 (R187) 2–3 mo	11,300	2	1.1	420.5	3	19	123
B186 (R189) 2–3 mo	11,300	3	1.8	322.3	2.3	12.3	102
B225 (R204) 2–3 mo	11,300	3	0.9	243.7	3.3	7	54.3
**Totals/Averages**	**45,200**	**17**	**1.2**	**271.5**	**2.6**	**10.2**	**111.5**

Summary of the ultrastructural characteristics of asymmetric NPY synapses across the ICc. Asymmetric synapses represented a minority of the NPY synapses (25 %) and mainly contained round vesicles.

**Table 3:** Summary of correlation coefficent *p*-values and Spearman’s rho values.

Symmetric - p values							
	Terminal area	Gold density	Postsynaptic Diameter	Active Zone Length	Vesicles at Active Zone	Total Vesicle Pool	Number of Mitochondria
Terminal area	NA	6.22E-15	0.507	0.6774	0.3977	9.85E-05	8.93E-05
Gold density	6.22E-15	NA	0.04752	0.7754	0.1518	0.001654	0.008535
Postsynaptic Diameter	0.507	0.04752	NA	0.9479	0.08943	0.5844	0.8292
Active Zone Length	0.6774	0.7754	0.9479	NA	6.06E-06	0.9644	0.4663
Vesicles at Active Zone	0.3977	0.1518	0.08943	6.06E-06	NA	0.4567	0.9158
Total Vesicle Pool	9.85E-05	0.001654	0.5844	0.9644	0.4567	NA	0.2558
Number of Mitochondria	8.93E-05	0.008535	0.8292	0.4663	0.9158	0.2558	NA
Asymmetric - p values							
	Terminal area	Gold density	Postsynaptic Diameter	Active Zone Length	Vesicles at Active Zone	Total Vesicle Pool	Number of Mitochondria
Terminal area	NA	7.76E-09	0.6808	0.4593	0.2299	0.0528	0.09537
Gold density	7.76E-09	NA	0.7343	0.4262	0.1543	0.05138	0.1494
Postsynaptic Diameter	0.6808	0.7343	NA	0.6387	0.6034	0.1491	0.8
Active Zone Length	0.4593	0.4262	0.6387	NA	1.08E-05	0.9787	0.2278
Vesicles at Active Zone	0.2299	0.1543	0.6034	1.08E-05	NA	0.5863	0.3272
Total Vesicle Pool	0.0528	0.05138	0.1491	0.9787	0.5863	NA	0.7614
Number of Mitochondria	0.09537	0.1494	0.8	0.2278	0.3272	0.7614	NA

Significant (p<0.05) positive correlation

Significant (p<0.05) negative correlation

Positive correlation

Negative correlation

## Data Availability

The data contained in the manuscript being submitted have not been previously published, submitted elsewhere or be submitted elsewhere while under consideration at Neuropeptides.

## Abbreviations:

FBN: Fischer Brown Norway
IC: inferior colliculus
ICc: central nucleus of the inferior colliculus
ICd: dorsal cortex of the inferior colliculus
IClc: lateral cortex of the inferior colliculus
LD: large dendrite
MD: medium dendrite
NPY: Neuropeptide Y
Pre NPY+: NPY positive presynaptic terminal
Pre NPY−: NPY negative presynaptic terminal
SD: small dendrite
